# Developing reliable hourly electricity demand data through screening and imputation

**DOI:** 10.1038/s41597-020-0483-x

**Published:** 2020-05-26

**Authors:** Tyler H. Ruggles, David J. Farnham, Dan Tong, Ken Caldeira

**Affiliations:** 1 Carnegie Institution for Science, Stanford, United States; 2 University of California, Irvine, Irvine, United States

**Keywords:** Scientific data, Energy supply and demand, Energy modelling, Statistics

## Abstract

Electricity usage (demand) data are used by utilities, governments, and academics to model electric grids for a variety of planning (e.g., capacity expansion and system operation) purposes. The U.S. Energy Information Administration collects hourly demand data from all balancing authorities (BAs) in the contiguous United States. As of September 2019, we find 2.2% of the demand data in their database are missing. Additionally, 0.5% of reported quantities are either negative values or are otherwise identified as outliers. With the goal of attaining non-missing, continuous, and physically plausible demand data to facilitate analysis, we developed a screening process to identify anomalous values. We then applied a Multiple Imputation by Chained Equations (MICE) technique to impute replacements for missing and anomalous values. We conduct cross-validation on the MICE technique by marking subsets of plausible data as missing, and using the remaining data to predict this “missing” data. The mean absolute percentage error of imputed values is 3.5% across all BAs. The cleaned data are published and available open access: 10.5281/zenodo.3690240.

## Abstract (summary)

**Table:** 

Measurement(s)	electricity demand validity
Technology Type(s)	computational modeling technique • Statistical Imputation • data screening
Factor Type(s)	electricity demand
Sample Characteristic - Location	contiguous United States of America

Machine-accessible metadata file describing the reported data: 10.6084/m9.figshare.12044736

## Background & Summary

Electricity system models typically require electricity usage (demand) as a known input. In July 2015, the U.S. Energy Information Administration (EIA) began collecting hourly electricity demand data from all 56 balancing authorities (BAs) across the contiguous United States (CONUS). BAs are responsible for the real-time balancing of electricity generation and demand within their territory and power interchange with surrounding BAs^1^. The EIA publishes demand data via an open access data portal: https://www.eia.gov/opendata/qb.php?category=2122628. This is the most temporally granular, publicly available demand data that covers all of CONUS.

Many BAs are missing hundreds to thousands of hours of demand data, this lack hampers the utility of this data for energy system modelers. We find that as of 10 September 2019, 2.2% of the hourly data in the EIA’s database are missing, and another 0.5% are either physically implausible (e.g. negative electricity usage) or suspicious for other reasons as outlined in the Methods section. Improving the usability of these data by screening out apparently erroneous data and producing continuous time series by gap-filling missing sections will be helpful to the energy modeling community.

**Fig. 1:**
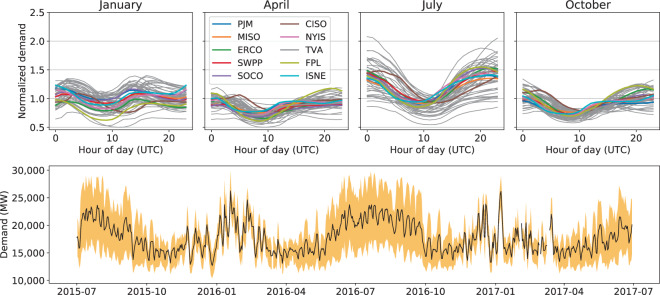
Demand profiles are shown based on screened EIA data. The top four panels show the typical daily profiles during four months for the 54 BAs retained in this analysis. The profiles show the mean value for each hour over the specified month. The ten BAs with the largest annual demand are shown in color. The bottom distribution shows two years of demand for the TVA BA. The black line denotes the mean daily demand value while the yellow shading shows the range between the maximum and minimum value for each day.

The demand data exhibit autocorrelation at sub-daily timescales and cyclic fluctuations on multiple time scales (daily to seasonal cycles). Figure 1 shows examples of normalized daily demand profiles for the BAs by month. The panels show that the profiles from BAs are often correlated with one another.

Each BA calculates their demand values and submits them to the EIA based on form EIA-930^2,3^. Demand ($D$) is derived as net generation within each BA territory ($NG$) minus total interchange ($TI$) with all connected BAs:

1 $$D=NG-TI.$$

Reported demand values can be affected by anomalies or missing values in either $NG$ or $TI$. Both $NG$ and $TI$ are aggregate values that result from a complex network of generators, tie line meters, and energy management and accounting systems^3^. According to the EIA, BAs occasionally report “anomalous data values involving blank, zero, negative, high, and low values”^4^. We exclude 2 of the 56 BAs from the analysis due to excessive irregularities. Eleven of the 54 remaining BAs have more then 1,000 missing hours during the past four years.

A variety of methods are used in the power industry to impute missing or anomalous data. Linear interpolation is a simple approach used when short intervals of missing data exist^5^. Nearest neighbor methods are commonly implemented when longer intervals of missing data exist^6^. The EIA uses a nearest neighbor method where values from the prior hour and day are used to replace missing or anomalous data.

In this Data Descriptor, we present a method to create complete time series data sets from a set of correlated demand records. We first developed an anomalous value screening process to flag the most extreme outliers. The algorithms were designed to incorporate the time series structure of the data and often use excessive deviations from continuity as a reason to flag a value.

We use a Multiple Imputation by Chained Equations (MICE) technique for imputation. Each demand value is predicted by using a linear regression on (a) the demand during that same hour for each other BA, (b) the one-hour leading and lagging demand values for the BA being predicted, and (c) the mean demand for that time of the year and hour of day for the BA being predicted. The values for (a) leverage cross-BA correlations to fill in data gaps, (b) encourage continuity, and (c) incorporate the influence of the mean daily and seasonal cycles for each BA.

The performance of the MICE technique is measured by intentionally marking plausible data as missing, imputing said data, and comparing these imputations against their true values. The mean absolute percentage error (MAPE) ranges from 1.3% to 11% across all BAs with a mean value of 3.5%. The imputation method demonstrates a small bias, which ranges from −0.38% to 4.0% depending on the BA with a mean value of 0.33%.

All of the data cleaning steps occur at the BA level to retain geographic resolution. The cleaned and validated data are available at 10.5281/zenodo.3690240. We aim to provide updated, cleaned data every 12 months to include recently published demand data and incorporate any available corrections to historical data.

## Methods

Society relies on the electric grid to provide reliable power that meets our immediate electricity demands. Common industry practices are to build excess supply into the electric system allowing grid operators to dispatch high-cost, infrequently used power plants to meet infrequent high demand events lasting a few hours at a time^7^. These infrequent high demand events have significant leverage in determining the structure of an electric system. Because of this leverage, entities such as the EIA are conserv ative in their determination of anomalous outliers in reported demand. They favor a cautious approach in categorizing and replacing anomalous values and choose to do so when aggregating demand data to produce regional and CONUS level hourly demand data. They keep the BA level hourly data “as-is”. The EIA classifies an hourly demand value as anomalous and marks it for imputation if the value is “missing or reported as negative, zero, or at least 1.5 times greater than the maximum of past total demand values reported by that BA”^3^.

The EIA uses a basic imputation process to replace the values marked as anomalous. The process relies on the preceding 24 hours of demand as well as forecasted demand data, which is also reported to EIA by the BAs. The imputation rules are as follows:

1. If a demand forecast exists for a marked hour, is not itself anomalous, and is from a BA with trustworthy forecast values, use that value
2. Otherwise, if a non-anomalous demand value exists for the previous hour, use that value
3. Otherwise, if a non-anomalous demand value exists for the same hour of the previous day, use that value
4. Otherwise, the marked value is effectively treated as a zero when aggregations are calculated

The EIA provides documentation for users including lists of known issues with the data^3,4^.

Within the EIA database, the missing hours are not spread evenly among the reporting BAs. The majority of missing hours are associated with the smaller and mid-sized BAs. Two of the smallest BAs, OVEC and SEC, have especially incomplete or anomalous data. The original OVEC BA was merged with the larger PJM BA on 1 December 2018, and therefore no longer exists nor reports data to EIA. The shorter OVEC data record is missing demand values for 34% of the hours between 2 July 2015 and 1 December 2018. Additionally, over 16% of the reported values are identical to that of the preceding hour. These streaks of identical demand values can last for hundreds of continuous hours indicating reporting problems. SEC has better initial coverage of hours with only 3% of hourly values missing. However, 13% of the hours have negative or zero values that make it difficult to describe the demand profile with confidence. Based on the reasonable looking data from both of these BAs, they each have mean demand less than 0.5 GW, while the CONUS mean demand is closer to 500 GW. Because of their relatively small sizes and excessive reporting irregularities, we remove these two BAs from this data cleaning process and all of the following analysis.

### Data acquisition

The EIA demand data that we cleaned and analyzed were queried from the EIA database using their Open Data portal through an application programming interface: https://www.eia.gov/opendata/. The data were queried on 10 September 2019, and span from 2015-07-01 05:00:00 UTC to 2019-08-31 23:00:00 UTC. The MICE process is tailored to expect full years of data. Because of this, we elect to analyze and clean the data spanning from 2015-07-02 00:00:00 UTC through 2019-07-01 23:00:00 UTC. The reported demand value for each time stamp corresponds to the integrated mean value in megawatts for the previous hour^2^. Demand is reported as integer values and corresponds to the 56 BAs listed in Supplementary Table S.4. As of July 2018, a few BAs provide subregional hourly demand data^8^. We do not currently use the subregional BA data because it would add substantial uncertainty into our imputation technique given that the July 2018 start of the subregional BA data does not align with the July 2015 start for the majority of other BAs.

As stated previously, we remove the SEC and OVEC BAs from this data cleaning process and all of the following analysis because of excessive reporting irregularities, missing data, and because OVEC was merged into another BA in 2018. This reduces the total from 56 BAs in the EIA database to 54 BAs which are fully analyzed.

There are two different types of missing data in the EIA database. For some hours, the EIA database queries return empty values for the hourly demand, which we mark as “EMPTY”. For some other hours, queries return no information, which we mark as “MISSING”. We treat both of these cases of missingness in the same way in our analysis and categorize both as ‘missing’ values. From the 54 analyzed BAs, 2.2% of the hourly values are categorized as ‘missing’. All demand values that are successfully retrieved (not ‘missing’) from the EIA database are originally categorized as ‘okay’.

### Anomalous value screening

We developed eight screening algorithms to flag anomalous values. These algorithms range from very simple, such as flagging negative values, to more complex algorithms that incorporate the local time series structure of the data into the screening process.

The screening is conducted in two steps. **Step 1** removes the most egregious anomalies where few or no calculations are needed. Afterward, in **Step 2**, the most extreme values have been removed making calculations of local characteristics of the data more reasonable. Through this screening process hourly values can be recategorized from ‘okay’ to other classifications based on the algorithms.

In many of the algorithms we use the median, rather than the mean, to describe the central tendency of the data. This is because the median of a data set is more robust against extreme outliers than the mean^9^. For a similar reason, we favor using the interquartile range (IQR) of the data as a description of its spread instead of using the standard deviation^9^. The IQR is defined as the range between the 25^th^ and 75^th^ percentile of a set of values. We often set selection thresholds as a multiplier of an IQR value. Because the IQR values are calculated for each BA, using multiples of the IQR values to set selection and rejection thresholds corresponds to screening or retaining data with similar characteristics across all BAs.

We denote ${d}_{t}$ as the hourly demand value reported in MW for hour $t$ for a given BA. Demand values that are screened are set to ‘NA’ in our code. For all of the calculations that follow, including calculations of the median and IQR values, ‘NA’ demand values are ignored. In cases where a derived value is illogical when calculated with a ‘NA’ input, such as the difference between subsequent demand values when one is ‘NA’ or the median of 10 ‘NA’ values, the derived quantity is ‘NA’. Derived ‘NA’ values are then ignored in subsequent calculations.

**Fig. 2:**
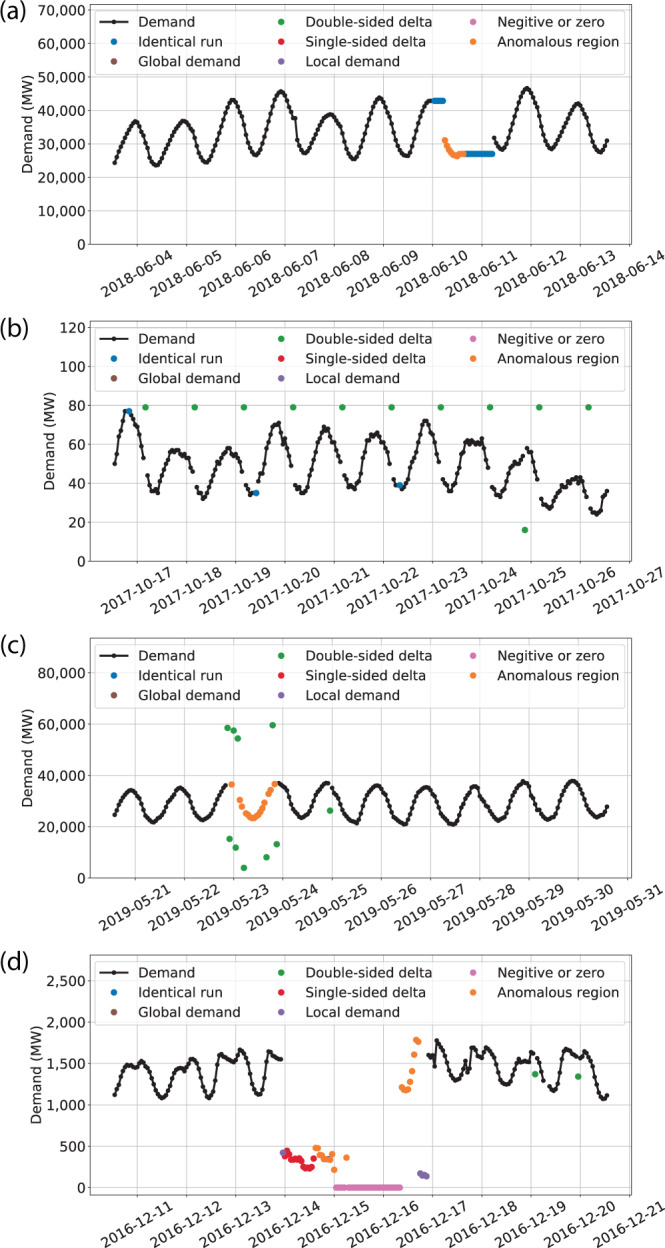
Illustration of several of the filtering algorithms. (**a**) The SWPP BA shows examples of the ‘identical run’ and ‘anomalous region’ algorithms. (**b**) The NSB BA demonstrates the ‘double-sided delta’ algorithm and a few falsely screened ‘identical run’ hours. (**c**) The SOCO BA demonstrates the ‘double-sided delta’ and ‘anomalous region’ filters. (**d**) The SCL BA demonstrates multiple screening algorithms with emphasis placed on the ‘single-sided delta’ filter and the ‘local demand’ filter.

We provide a description of the eight screening algorithms below. In the following descriptions, the word “local” is used temporally. Additional details are included in the Supplemental Material (see the **Anomalous value screening details** section). Figure 2 illustrates examples of the screening algorithms. The algorithms are as follows:

**Step 1:**

1. ‘Negative or zero’ filter: Flag negative and zero values.
2. ‘Identical run’ filter: In a series of identical values, flag the third hour onwards as anomalous.
3. ‘Global demand’ filter: Flag extreme high-magnitude outliers to make further calculations more reasonable. For each BA, we screen values that are at least 10 times larger than the four year median demand value.
4. ‘Global demand ± 1 hour’ filter: Flag hours immediately surrounding ‘global demand’ filtered hours because they often have large deviations that do not meet the threshold of the ‘global demand’ filter.

**Step 2:**

1. ‘Local demand’ filter: Construct an estimated demand value for each hour and each BA based on a local 48 hour moving median and the typical local daily cycle. If ${d}_{t}$ deviates significantly from the estimate, flag that hour. Significance is defined with respect to the local IQR of the difference between demand and the 48 hour moving median (IQR of the spread of the daily cycle).
2. ‘Double-sided delta’ filter: Flag single hours that appear to be erroneous spikes in the demand. The threshold for determining spikiness is defined with respect to the change in demand from hour to hour. The threshold is locally quantified for each BA using an IQR based on the hourly differences between subsequent demand values.
3. ‘Single-sided delta’ filter: Flag sequences of hours that significantly deviate from both a continuous demand profile and expectations. Demand expectations are calculated similar to those for the ‘local demand’ filter. To calculate the deviations from a continuous demand profile we use the IQR values calculated for the ‘double-sided delta’ filter.
4. ‘Anomalous region’ filter: Flag remaining ‘okay’ demand values in the middle of chaotic sections of data. This catches un-flagged residual anomalous values that are often present within highly chaotic regions.

The eight different screening algorithms each serve a unique purpose and are applied in the order enumerated above. Table 1 shows the quantity of hours screened by each algorithm.

**Table 1:** The number of hours assigned to each category after the screening process is shown.

Category	Counts	Percent (%)
‘okay’	1,843,424	97.36
‘missing’	41,015	2.16
‘negative or zero’	863	0.05
‘identical run’	3,861	0.20
‘global demand’	170	0.01
‘global demand ± 1 hour’	285	0.01
‘local demand’	671	0.03
‘double-sided delta’	1,212	0.06
‘single-sided delta’	181	0.01
‘anomalous region’	1,773	0.09
Flagged for Imputation	50,031	2.64
Total Hours	1,893,456	100.00

### Multiple Imputations through Chained Equations (MICE)

The Multiple Imputations through Chained Equations (MICE) framework is essentially a series of linear regressions run in sequence and repeated until the estimated values for missing values have sufficiently converged. In this case we impute the hourly demand for each BA. To do this, we first develop a set of predictor variables and a set of regression equations (outlined in the *MICE variables* and *MICE equations* sections). Finally, we conduct the MICE method as outlined in the *MICE algorithm* section below. Throughout this section we consider all values categorized as ‘missing’ or flagged as anomalous to be ‘missing’ since both of these types of hourly demand observations are treated identically in the MICE procedure.

#### MICE variables

We conduct MICE in the log space (*d*′ from Eq. 2) since log-transformed hourly demand data are closer to being normally distributed and our MICE framework assumes normality of regression errors. That is, the hourly demand data are transformed into the log space before the derivation of the MICE predictors and completion of the MICE algorithm.

2 $$d{\prime} =lo{g}_{e}(d)$$

The following variables are used in our MICE procedure.

${d}_{r,t}^{{\rm{{\prime} }}}$ The log-space hourly demand for hour $t$ and BA $r$. This variable includes missing data in our data set.

${{\boldsymbol{d}}}_{{\boldsymbol{-r}},{\boldsymbol{t}}}^{{\rm{{\prime} }}}$ The vector of hourly demand values for hour $t$ and all non-$r$ BAs. This variable includes missing data in our data set.

${d}_{r,\mathrm{t-1}}^{{\rm{{\prime} }}}$ The hourly demand for hour $t-1$ and BA $r$. This variable includes missing data in our data set.

${d}_{r,\mathrm{t+1}}^{{\rm{{\prime} }}}$ The hourly demand for hour $t+1$ and BA $r$. This variable includes missing data in our data set.

$C({d}_{r,t}^{{\rm{{\prime} }}})$ The mean demand for the day of year and hour of day associated with hour $t$ for BA $r$. $C({d}_{r,t}^{{\rm{{\prime} }}})$ is computed separately for each of the 24 hours of the day and is the mean of the temporally closest 60 values for BA $r$ for the same hour of day as time step $t$. Missing values in this interval are ignored (i.e. if there are 10 missing values out of 60, then $C({d}_{r,t}^{{\rm{{\prime} }}})$ is the mean of 50 values). Since there are four years of data in this original data set, this variable is the mean of plus or minus 1 week of data. For example, the value of $C({d}_{r,t}^{{\rm{{\prime} }}})$ for 2 PM on 10 May 2016 for the CISO BA would be the mean of all 2 PM demand values for CISO between May 3^rd^ and May 17^th^ for all years 2016 through 2019.

#### MICE equations

**Fig. 3:**
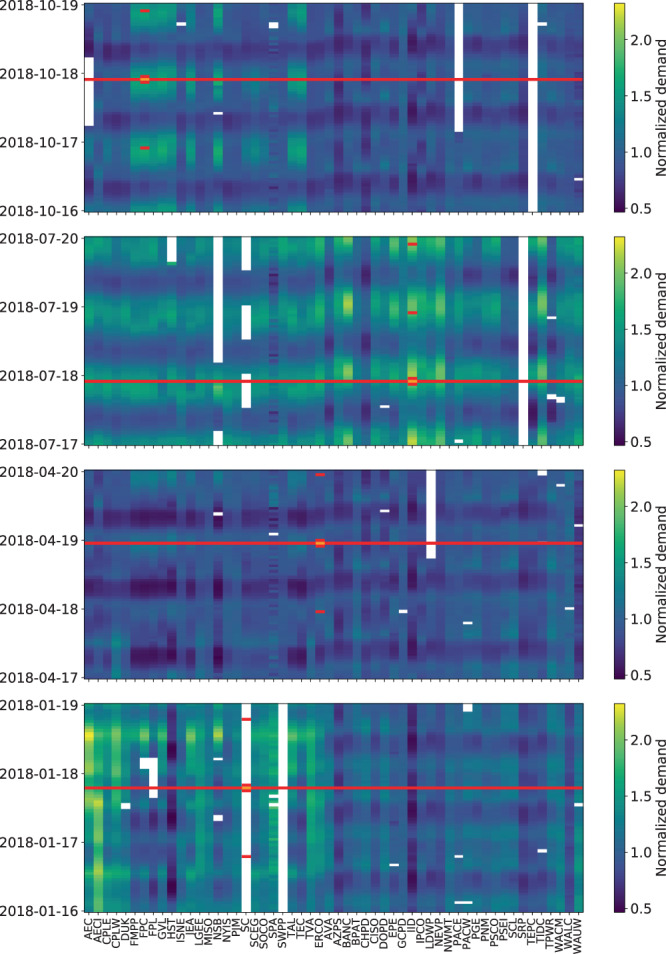
The normalized hourly demand values for the 54 BAs are shown for four different three-day time slices, one from each season. The empty (white) cells were either ‘missing’ or flagged as anomalous. In each panel of the figure, there is an example of the predictor values (red cells) that would be used to predict a given demand value (orange cell). The three days shown are all Tuesday, Wednesday, and Thursday and end on the first hour of Friday UTC. The heatmap color range is scaled the same for all seasons to show the seasonal trends. The daily cycle is visible as well.

We predict $d{{\prime} }_{r,t}$ using the 1-hour lagging ($d{{\prime} }_{r,t-1}$) and leading ($d{{\prime} }_{r,t+1}$) variables for BA $r$, the vector of hour $t$ demand from all other BAs (${{\boldsymbol{d}}}_{{\boldsymbol{-}}{\bf{r}}{\boldsymbol{,}}{\boldsymbol{t}}}^{{\rm{{\prime} }}}$), and the day of year and hour of day mean value for BA $r$ ($C(d{{\prime} }_{r,t})$; see Eq. 3). The leading and lagging variables have predictive capacity because the lag-1 correlation of each BA is generally high (e.g., Figs. 2 and 3). The hour $t$ demands at other BAs have predictive capacity because the cross-BA correlations are substantial in many cases (Fig. 3). Lastly, the day of year and hour of day mean value for a BA adds stability to the model when long temporal gaps exist for a BA. We use all complete observations to estimate the parameters ${\alpha }_{r,1}$, ${\alpha }_{r,2}$, ${\beta }_{{\boldsymbol{-}}{\boldsymbol{r}}}^{{\boldsymbol{c}}{\boldsymbol{o}}{\boldsymbol{n}}{\boldsymbol{c}}}$, and ${\gamma }_{r}$ via Eq. 3.

3 $${d}_{r,t}^{{\rm{{\prime} }}}={\alpha }_{r,1}{d}_{r,\mathrm{t-1}}^{{\rm{{\prime} }}}+{\alpha }_{r,2}{d}_{r,\mathrm{t+1}}^{{\rm{{\prime} }}}+{\beta }_{{\boldsymbol{-}}{\boldsymbol{r}}}^{{\boldsymbol{c}}{\boldsymbol{o}}{\boldsymbol{n}}{\boldsymbol{c}}}{\boldsymbol{d}}{{\rm{{\prime} }}}_{{\boldsymbol{-}}{\boldsymbol{r}}{\boldsymbol{,}}{\boldsymbol{t}}}+{\gamma }_{r}C({d}_{r,t}^{{\rm{{\prime} }}})+{\epsilon }_{r,t}^{conc}$$

We predict ${d}_{r,\mathrm{t-1}}^{{\rm{{\prime} }}}$ and ${d}_{r,\mathrm{t+1}}^{{\rm{{\prime} }}}$ using the corresponding vectors of hourly demand values from all other BAs (${{\boldsymbol{d}}}_{{\boldsymbol{-}}{\bf{r}}{\boldsymbol{,}}{\boldsymbol{t}}{\boldsymbol{-}}{\boldsymbol{1}}}^{{\rm{{\prime} }}}$ and ${{\boldsymbol{d}}}_{{\boldsymbol{-}}{\bf{r}}{\boldsymbol{,}}{\boldsymbol{t}}{\boldsymbol{+}}{\boldsymbol{1}}}^{{\rm{{\prime} }}}$; Eqs. 4 and 5). We do not use leading and lagging variables because their inclusion leads to feedbacks that markedly slow convergence of the imputed variables. We use all complete observations to estimate the parameters ${\beta }_{{\boldsymbol{-}}{\boldsymbol{r}}}^{{\boldsymbol{l}}{\boldsymbol{a}}{\boldsymbol{g}}}$, and ${\beta }_{{\boldsymbol{-}}{\boldsymbol{r}}}^{{\boldsymbol{l}}{\boldsymbol{e}}{\boldsymbol{a}}{\boldsymbol{d}}}$ via Eqs. 4 and 5.

4 $${d}_{r,t-1}^{{\rm{{\prime} }}}={\beta }_{{\boldsymbol{-}}{\bf{r}}}^{{\bf{l}}{\bf{a}}{\bf{g}}}{{\boldsymbol{d}}}_{{\boldsymbol{-}}{\boldsymbol{r}}{\boldsymbol{,}}{\boldsymbol{t}}{\boldsymbol{-}}{\boldsymbol{1}}}^{{\rm{{\prime} }}}+{\epsilon }_{r,t-1}^{lag}$$

5 $${d}_{r,t+1}^{{\rm{{\prime} }}}={\beta }_{{\boldsymbol{-}}{\bf{r}}}^{{\bf{l}}{\bf{e}}{\bf{a}}{\bf{d}}}{{\boldsymbol{d}}}_{{\boldsymbol{-}}{\boldsymbol{r}}{\boldsymbol{,}}{\boldsymbol{t}}{\boldsymbol{+}}{\boldsymbol{1}}}^{{\rm{{\prime} }}}+{\epsilon }_{r,t+1}^{lead}$$

We do not define a regression with $C({d}_{r,t}^{{\rm{{\prime} }}})$ as the predictand because $C({d}_{r,t}^{{\rm{{\prime} }}})$ does not include any missing values by construction.

#### MICE algorithm

First, we define an order for the regressions (Eqs. 3, 4 and 5) to take place. We conduct the regressions in the following sequence: 1) predict all missing $d{{\prime} }_{r,t}$ in the alphabetical order of the BAs, 2) predict all missing $d{{\prime} }_{r,t-1}$ in the alphabetical order of the BAs, 3) predict all missing $d{{\prime} }_{r,t+1}$ in the alphabetical order of the BAs (see Supplementary Table S.4 for the alphabetical ordering).

The MICE algorithm then proceeds through the following 4 steps for each of $n$ chains (or realizations) of the MICE algorithm. In our case, we use 16 chains.

**Step 1:** Initialize all missing values with random draws from the observed data.

**Step 2:** For each variable ${d}_{r,t}^{{\rm{{\prime} }}}$, ${d}_{r,\mathrm{t-1}}^{{\rm{{\prime} }}}$, and ${d}_{r,t+1}^{{\rm{{\prime} }}}$, fit a regression model by Eqs. 3, 4 and 5, in the order specified above. Specifically, update the estimate for each of the missing values for each variable in the sequence outlined above. Start with Eq. 3 with the AEC BA as the predictand. Fit this regression using all observations for which AEC is non-missing. Next, use this fit to predict all missing values of AEC. Retain these estimates for AEC and move onto the AECI BA. Continue until you have completed all regressions described above.

**Step 3:** Repeat **Step 2** until estimates for the missing values have converged. In our case, we complete 10 iterations for each of the 16 chains.

**Step 4:** For each imputed hour, take the mean of the 16 chains (i.e imputation realizations) and use this mean as the estimate.

**Fig. 4:**
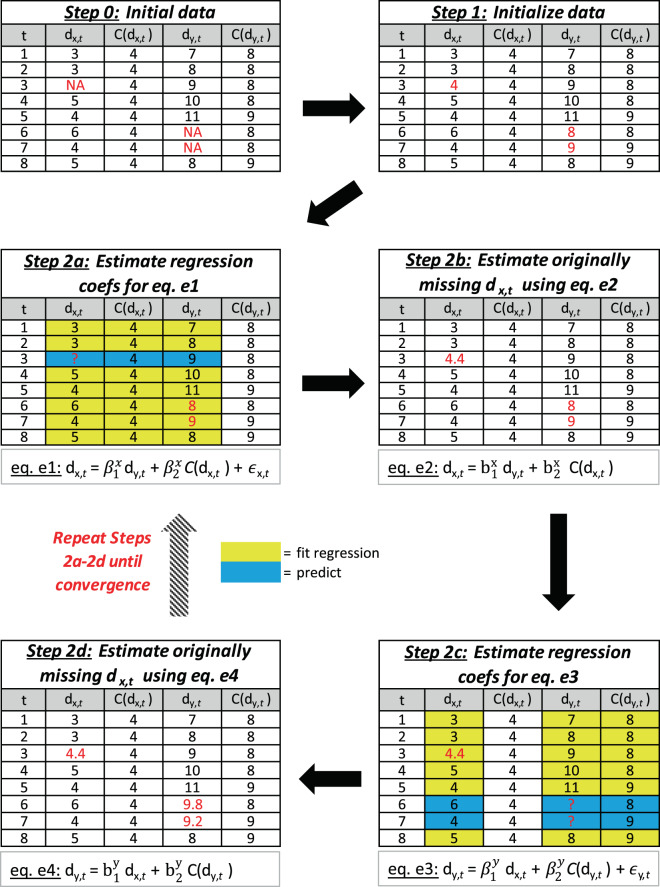
Example of the MICE framework.

This process is illustrated in Fig. 4 for a simplified example of two BAs (${d}_{x,t}$ and ${d}_{y,t}$), each with two predictors: their seasonal mean ($C({d}_{x,t})$ and $C({d}_{y,t})$) and the other BA’s demand.

We use the *mice* package^10^ in the R statistical computing programming language^11^. Convergence of the chains is measured via visual inspection of the mean and standard deviation of each BA’s hourly demand data. We use a normal bootstrapping approach, which assumes normality of regression residuals and fits the linear regressions on bootstrapped samples of the data.

## Data Records

The complete and cleaned data sets are published in Zenodo (ref. ^18^) with the corresponding DOIs:

• v1.1 (specific version): 10.5281/zenodo.3690240

• Concept (all versions): 10.5281/zenodo.3517196

The data are published under the Creative Commons Attribution 4.0 International license. The published data span from the first full day of data collection on 2015-07-02 00:00:00 UTC to 2019-07-01 23:00:00 UTC.

## Technical Validation

We assess the performance of the anomaly screening and MICE method in multiple ways. In the **Second anomaly screening** section we rerun the anomaly identification code on the data output from the MICE process. This evaluates whether the imputed values trigger the original anomaly identification algorithms. Second, in the **Imputing plausible data** section, we remove ‘okay’ hourly values from the data after the anomaly screening process to create extra gaps for imputation. This allows a comparison of the imputed values against observed demand values that are not flagged as anomalous. Finally, in the **Energy system modeling** section, we run a capacity and dispatch-type energy model with different input demand time series data and compare the resulting least-cost system configurations. This last validation step is designed to understand some of the practical implications of our demand imputation scheme for energy system optimization models.

### Second anomaly screening

We are able to see both the quality of the anomaly screening and the imputation method by running the imputed data through the anomaly screening process a second time. Over 85% of the hours that are flagged on a second screening are flagged by the ‘identical run’ filter. These instances mainly occur for small BAs with mean demand values less than 300 MW. For these small BAs there is a reasonable chance of finding three hours with the same integer demand value during the peaks and troughs of the daily cycle. We ignore the hours selected by the ‘identical run’ filter and sort the remaining hours that are screened on a second pass into three categories:

**Category 1:** The imputed values are nearly identical to their initial values indicating imperfect screening algorithms (11 hours found).

**Category 2:** The imputed values appear significantly improved from their original anomalous values allowing new anomalous structures in the data to be found (57 hours found). This happens when the screening criteria does not remove all of the anomalous structure on the initial screening.

**Category 3:** The imputed values appear improved from their original anomalous values. But, because of the surrounding structures, the imputed values are themselves flagged as new anomalies (44 hours found).

The 11 hours in **Category 1** all result from cases where the ‘local demand’ filter was overly aggressive in flagging data in the peaks or troughs of the daily cycle. This happens when there were large, rapid weather changes and the 48 hour moving median (${M}_{t,48{\rm{hr}}}$ as defined in the supplemental material) does not capture the changes. The reason the imputed values align so well with the original values in these cases is because the 1-hour leading and lagging values (${d}_{r,t-1}^{{\prime} }$ and ${d}_{r,t+1}^{{\prime} }$) in the imputation model emphasize data continuity. Therefore, the imputed values are nearly identical to the original values. We are not concerned about these few cases.

We find 57 hours fitting the **Category 2** description. These 57 hours are the result of an imperfect screening process where we balanced screening as many outliers as possible against falsely flagging hours for imputation. The 57 hours are significantly less than the 9,016 hours screened as anomalous on the initial pass showing a substantial improvement in data quality. Two examples of **Category 2** can be seen in Supplementary Fig. S.8.

During the second screening, 44 hours are marked anomalous comprising three anomalous events within the data contributing to **Category 3**. These are the only cases where imputed values are flagged during the second screening. The three cases can be seen in Supplementary Fig. S.9, which shows that one of the events contributes 37 of the 44 hours.

The results of the second anomaly screening test are summarized in Table 2. We anticipate that future analysts will be interested in combining the BA level data into larger regional totals. Because of this, we additionally report results of the second anomaly screening process after combining the BA level data into the 13 EIA defined regions (see mapping in Supplementary Table S.4). In this case, all initial screening and imputation is still completed at the BA level. Only afterwards are the hourly values summed into regional totals. The second anomaly screening is then run on these regional aggregates. In this case, nearly all of the second round anomalies are smoothed out via aggregating. Only a single hour is screened for the 13 regions.

**Table 2:** The total number of hours falling into each category is shown.

Screening Algorithm	First Screening: 54 BAs		Second Screening: 54 BAs	
	Counts	Percent (%)	Counts	Percent (%)
‘okay’	1,843,424	97.36	1,892,736	99.962
‘missing’	41,015	2.16	0	0.000
‘negative or zero’	863	0.05	0	0.000
‘identical run’	3,861	0.20	608	0.032
‘global demand’	170	0.01	0	0.000
‘global demand ± 1 hour’	285	0.01	0	0.000
‘local demand’	671	0.03	23	0.001
‘double-sided delta’	1,212	0.06	28	0.001
‘single-sided delta’	181	0.01	15	0.001
‘anomalous region’	1,773	0.09	46	0.002
Flagged for Imputation	50,031	2.64	720	0.038
Flagged for Imputation: Ignore Identical Runs	46,170	2.50	112	0.006
Total Hours	1,893,456	100.00	1,893,456	100.00

The second round of screening, after the initial screening and imputation, shows a vast reduction in the quantity of hours flagged as anomalous. This reduction indicates strong anomaly screening and imputation performance.

This same regional exercise is repeated for the three CONUS interconnects: Eastern, Western, and Texas. The interconnect results exclude contributions from utilities in Mexico and Canada. At the interconnect level, zero second round anomalies are identified. The results of these tests at the BA, regional, and interconnect level demonstrate a vast improvement over the original raw, “as-is” BA data.

### Imputing plausible data

After running the anomalous value screening process, we record the length of each continuous region marked for imputation. These continuous regions can be a combination of ‘missing’ and screened values. We then select random locations in the data to remove ‘okay’ data. We remove data with the same gap lengths and number of gaps as those created by the original ‘missing’ and screened values. This provides additional gaps where the data are known and have been classified as ‘okay’, but are marked for imputation in order to understand the efficacy of the imputation model. Sixteen new data sets are created with additional gaps. The 16 new data sets provide a large randomized set of hourly values for imputation and comparison. Each of the 16 new data sets is run through the MICE process using 16 chains each, identical to the MICE process we previously used.

**Fig. 5:**
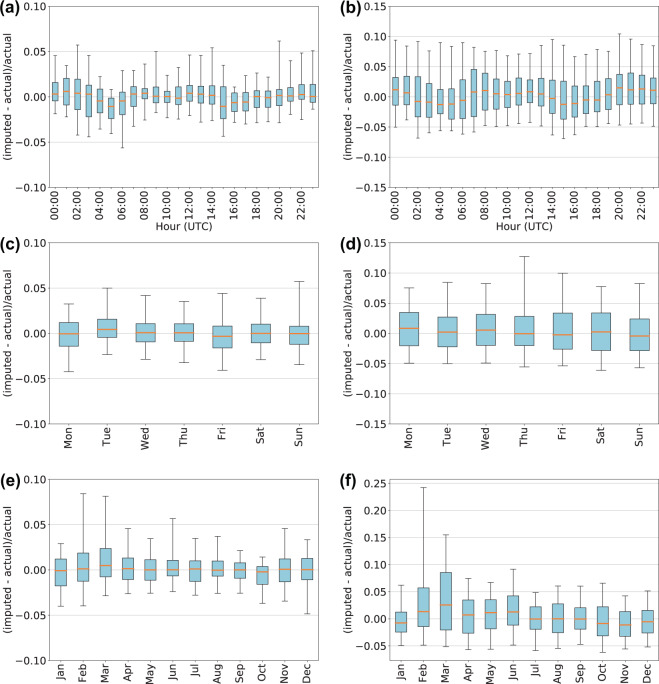
Box plots compare imputed values against the observed hourly values. The box plots highlight different temporal characteristics on the x-axes. Panels (a, c and e) show short imputation gaps while panels (b, d and f) show longer imputation gaps.

**Fig. 6:**
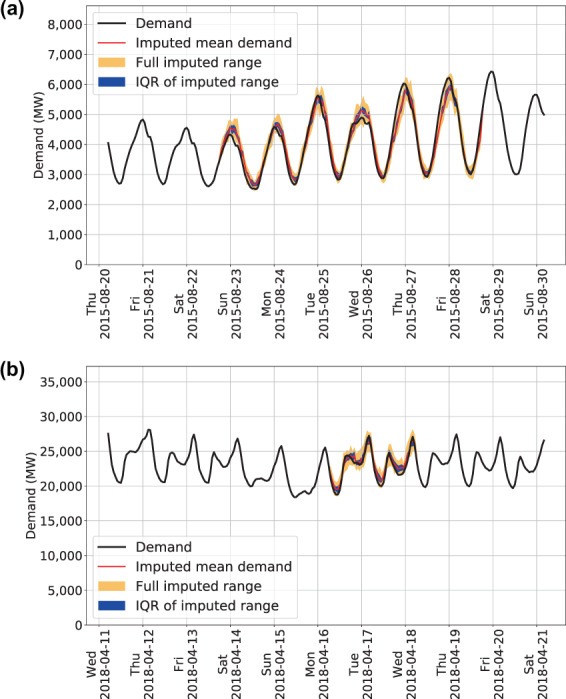
Time series distributions of the original values compared against the imputed values. Black lines represent the ‘okay’ observed values. Red lines represent the imputed values. The blue band shows the IQR of the 16 imputation chains for each imputed hour, while the extent of the yellow band shows the maximum and minimum range of the 16 imputation chains. Panel (a) shows the LDWP BA and panel (b) shows the CISO BA.

The resulting imputed values are compared in multiple ways. Box plots are used to visualize trends or deviations in the imputed values from the observed values. For example, to ensure that there are not substantial biases by hour of day or month of year. Examples of these diagnostic plots can be seen in Fig. 5 for the CISO BA. The data are grouped into two categories based on the length of the imputed gaps: gaps of one or two consecutive imputed hours, where the imputed values are adjacent to at least one ‘okay’ value. And, gaps of three or more consecutive imputed hours where at least one hour is fully surrounded by other imputed values. The time series structure of the imputed data can be seen in Fig. 6 showing distributions of the original ‘okay’ observed data with the imputed data overlaid. The time series structure is clearly preserved with the imputation method.

The mean absolute percentage error (MAPE) (Eq. 6) is calculated from all imputed hours that correspond to a known ‘okay’ demand value. ${A}_{t}$ is the actual observed value and ${I}_{t}$ is the imputed value.

6 $${\rm{MAPE}}=\frac{100 \% }{n}\mathop{\sum }\limits_{t=1}^{n}\left|\frac{{A}_{t}-{I}_{t}}{{A}_{t}}\right|$$

We also calculate the mean percentage error (Eq. 7) to quantify the bias in the MICE method.

7 $${\rm{mean}}\,{\rm{percentage}}\,{\rm{error}}=\frac{100 \% }{n}\mathop{\sum }\limits_{t=1}^{n}\frac{{A}_{t}-{I}_{t}}{{A}_{t}}$$

We split the results similarly as above into a short category (gaps of 1 or 2 imputed hours) and a long category (gaps of 3+ imputed hours). In addition, we provide an inclusive MAPE value for all gaps calculated together. The values are documented in Table 3. The MAPE for the imputed values measured inclusively ranges from 1.3% to 11% with a mean value across all BAs of 3.5%. The mean percentage error for the imputed values measured inclusively ranges from −0.38% to 4.0% with a mean value across all BAs of 0.33% indicating minimal bias in the imputation method.

**Table 3:** Imputation results are reported for each BA including the mean percentage error and MAPE.

BA	Median dem. (MW)	Imputed hours	Mean pct. error (%)	MAPE (%)			Forecast MAPE_*F*_ (%)
				Short	Long	Inclusive	
PJM	88,679	102	0.179	1.42	1.98	1.91	3.59
MISO	72,912	25	0.237	1.08	1.77	1.34	2.72
ERCO	38,965	240	0.586	3.77	5.32	5.22	2.53
SWPP	28,546	1,307	−0.071	1.61	2.37	2.34	3.02
SOCO	25,407	1,855	−0.039	1.79	2.38	2.36	3.88
CISO	25,154	349	−0.033	1.66	3.05	2.74	3.04
NYIS	17,812	28	−0.295	0.88	2.33	2.13	3.49
TVA	17,173	752	−0.022	1.51	2.40	2.36	2.15
FPL	13,905	177	0.246	1.61	2.51	2.18	5.29
ISNE	13,754	66	−0.106	1.33	2.65	1.49	2.76
DUK	11,365	240	0.226	2.15	2.62	2.59	2.78
CPLE	6,585	229	0.154	1.96	3.03	2.88	3.81
BPAT	6,203	54	0.232	1.15	2.24	1.86	2.00
FPC	5,722	212	0.019	1.17	2.20	1.93	20.85^†^
PACE	5,382	3,604	0.033	2.36	3.57	3.39	4.81
PSCO	4,905	526	0.093	1.53	3.58	3.49	3.59
LGEE	3,939	101	0.156	2.04	2.89	2.78	5.44
NEVP	3,829	890	0.129	2.45	2.99	2.86	3.51
PSEI	3,367	242	0.754	2.53	3.54	3.47	49.97^†^
AZPS	3,181	152	0.103	1.94	3.28	3.07	3.81
LDWP	3,126	585	0.423	2.37	4.60	4.45	5.06
SRP	2,998	3,358	0.009	1.96	2.87	2.79	5.15
WACM	2,883	909	−0.062	1.56	4.98	4.36	4.05
SC	2,666	3,357	−0.383	2.73	3.49	3.47	4.03
SCEG	2,621	26	0.001	2.06	2.33	2.30	2.96
PGE	2,349	786	0.739	1.67	3.25	3.21	2.22
TEC	2,333	27	0.547	1.14	2.59	2.37	4.57
PACW	2,298	4,039	0.402	3.23	4.33	4.25	6.34
AECI	2,240	13	0.548	2.82	3.21	2.86	5.11
FMPP	1,969	671	0.162	1.60	1.87	1.84	5.17
BANC	1,889	1,478	−0.070	2.15	3.81	3.78	3.13
IPCO	1,826	258	0.045	2.47	5.36	4.01	5.74
PNM	1,545	53	0.239	1.12	2.81	2.08	2.93
TEPC	1,532	3,838	1.727	4.82	9.32	9.25	13.97^†^
JEA	1,420	241	0.912	2.05	4.32	3.95	6.46
AVA	1,351	445	0.247	2.79	3.52	3.38	9.24^†^
NWMT	1,301	152	0.110	1.34	3.12	2.61	3.58
SCL	1,103	198	0.317	1.58	3.19	2.75	2.15
WALC	1,020	663	0.615	4.52	7.09	6.36	20.13
EPE	886	98	0.062	1.61	2.99	1.72	3.50
GCPD	568	171	0.220	0.88	3.96	1.34	2.56
CPLW	557	237	0.165	2.11	4.56	3.78	3.77
TPWR	541	305	0.148	1.39	2.25	2.05	2.86
AEC	478	304	0.285	2.51	4.75	4.46	93.73^†^
IID	351	742	4.033	7.80	11.27	10.88	11.03
TAL	301	46	0.121	1.73	“—”	1.73	4.32
TIDC	282	386	−0.035	1.64	3.44	2.88	2.82
GVL	217	3,308	0.144	2.48	3.66	3.60	8.58^†^
CHPD	198	205	1.142	3.13	12.67	6.96	123.41
DOPD	172	1,658	0.036	1.94	5.06	3.63	9.15
WAUW	90	4,778	0.664	4.51	7.99	7.58	6.49
SPA	70	293	1.017	8.12	9.14	8.33	11.72^†^
HST	62	1,665	0.798	2.47	5.29	4.34	7.44
NSB	45	3,587	−0.069	3.50	5.44	4.97	8.80

For comparison, the MAPE of the forecast values versus the actual values is also included. The MAPE values are categorized into three columns: short gaps of 1 or 2 hours, long 3+ hour gaps, and all gaps inclusive. The BAs are ordered by their median demand. The total number of ‘missing’ and screened hours for each BA is denoted in the ‘Imputed Hours’ column. There are no long gaps for the TAL BA as noted by the “—”.

^†^BAs where forecast and reported demand values are expected to disagree based on EIA guidance.

These imputation-based MAPE values can be compared against an alternative MAPE (MAPE_*F*_) where the imputed values (${I}_{t}$) in Eq. 6 are replaced with the 24-hour day ahead forecast values (${F}_{t}$). These ${F}_{t}$ are provided by each BA and are retrievable from the EIA database. We include values in the calculation of MAPE_*F*_ if ${A}_{t}$ exists and is categorized as ‘okay’ and ${F}_{t}$ exists. This is not a perfect comparison because the compared hours are not identical between the two methods. However, the results allow a rough comparison of the imputed MAPE versus MAPE_*F*_. The MAPE_*F*_ values range from 2.00% to 123.41% with a median value of 10.10%. The EIA states: “for some BAs, where a significant portion of their demand is outside their system or other BAs control a significant amount of demand inside their system, the comparison between actual and forecast demand is not very meaningful”^3^. The list of BAs for which this is the case includes: AEC, AVA, FPC, GVL, PSEI, SPA, and TEPC and are marked in Table 3. The MAPE_*F*_ values show that the developed imputation algorithm performs as well or better than the average 24-hour day ahead forecast for most BAs. It is worth noting that in many instances of missing demand data in the EIA database, the forecast values are also missing. Therefore, relying on forecasts to impute demand values does not work in all situations.

### Energy system modeling

The goal of creating non-missing, continuous, and physically plausible demand data time series is to facilitate energy systems analyses. Erroneous, non-plausible, and missing values can have a large impact on model results. We use a single node capacity and dispatch-type model to demonstrate this. The model uses linear optimization to solve for the least-cost electricity generation mix for different energy technologies and ensures that the technologies can be deployed to satisfy 100% of electricity demand. The energy technologies that we include are: natural gas (NG) and nuclear power plants, wind and solar renewable resources, and generic energy storage, whose cost is based on lithium-ion battery storage. The capital, operations and maintenance, fuel, and other variable costs for these technologies are all taken from the EIA^19,20^. We run the model over our three calendar years of complete demand data: 2016, 2017, and 2018. Corresponding hourly resolution wind and solar capacity factors are derived for these three years for each geographic region using the MERRA-2^21^ reanalysis data set.

To compare model results we prepare the input demand data using three data cleaning techniques:

**Method 1:** Use raw values from the EIA database at the BA level with negative and missing demand values set to zero.

**Method 2:** Use the results of the EIA anomaly identification and imputation methods.

**Method 3:** Use the anomaly identification method defined in this Data Descriptor with missing and anomalous values imputed using the MICE method described on page 5.

We analyze results at two levels of geographic granularity. One level aggregates all BAs for a CONUS total. The other level splits the BAs according to the three CONUS interconnects: Eastern, Western, and Texas. We use regional and coarser analyses because the results of the EIA anomaly identification and imputation method are published at the regional level but not at the BA level.

Summary statistics are shown in Supplementary Table S.5 and include the mean, maximum, and minimum hourly demand for these different data cleaning methods. A strong case for data cleaning is made when comparing the excessively large maximum demand values for **Method 1** in the Western and CONUS regions. Noticeable differences are seen between **Method 2** and **Method 3** comparing the minimum demand values for Texas because the EIA imputation method (**Method 2**) lacks the ability to fill in long consecutive demand gaps.

The optimized system capacities are calculated for each region, for each technology case, and for each data cleaning method and are shown in Supplementary Table S.6. The least-cost system with all the mentioned technologies included, based on current EIA costs, usually results in a system composed of purely natural gas with zero storage capacity. This is the simplest scenario for comparisons; because of the 100% reliability requirement these systems are sized according to the peak demand hour over the 3 years of input data. The large maximum demand values for the Western and CONUS regions, when using minimal data cleaning, are reflected in the large capacities for **Method 1** in Supplementary Table S.6. The differences between **Method 2** and **Method 3** are smaller and range from some capacities with perfect alignment, many with deviations less than 5%, and one difference over 10% (compare the wind capacities for the Western region under the no NG scenario). The capacities for Texas are nearly identical for all data cleaning cases showing the value of high-quality initial data.

Overall, the model results from the data cleaning comparison shown in Supplementary Table S.6 demonstrate reasonable agreement between the EIA method and the method developed in this Data Descriptor. The areas where our method proves most beneficial are 1) for analysts interested in using non-missing, continuous, and physically plausible demand data at the BA level, and 2) in cases where there are multi-day missing sections of demand data reported to the EIA. As shown previously, the MICE imputation method is capable of predicting reasonable demand data during multi-day gaps.

## Usage Notes

The complete and cleaned data can be used in the same manner as one would use the original EIA hourly demand data. We expect the primary use of this data will be energy system modeling where hourly temporal granularity is needed. A brief discussion of how the data can be used in an energy system model is provided in the **Energy system modeling** section on page 10. The data we publish bring the significant benefit of imputing at the BA level and providing complete and cleaned records at this geographic granularity. This allows analysts to pursue more geographically detailed studies, rather than limit analysis to the level of the 13 EIA regions.

To create a simple time series distribution of the demand for the ERCOT BA, follow these commands after downloading and unzipping the file “EIA_Cleaned_Hourly_Electricity_Demand_Data-v1.1.zip” from ref. ^18^. Make sure that the python libraries *pandas* and *matplotlib* have been previously installed.

$ cd PATH/TO/UNZIPPED/DIRECTORY # base of the unzipped directory

$ python -i

>>> import pandas as pd

>>> import matplotlib.pyplot as plt

>>> file_name = ‘data/release_2019_Oct/balancing_authorities/ERCO.csv’

>>> df = pd.read_csv(file_name, na_values=[‘MISSING’, ‘EMPTY’])

>>> df[‘date_time’] = pd.to_datetime(df[‘date_time’])

>>> fig, axs = plt.subplots(2)

>>> axs[0].plot(df[‘date_time’], df[‘cleaned demand (MW)’])

>>> axs[1].plot(df.loc[1000:1250, ‘date_time’], df.loc[1000:1250,

… ‘cleaned demand (MW)’])

>>> plt.show()

>>> exit()

## Supplementary information

Supplementary Information

## Data Availability

The code used to clean and analyze the data is published in Zenodo (ref. ^12^) with the corresponding DOIs: • v1.1 (specific version): 10.5281/zenodo.3737085 • Concept (all versions): 10.5281/zenodo.3678854 The code is available under the MIT License. The repository contains four notebooks that reproduce the following workflow: **Step 1:** Query the EIA database for raw demand data **Step 2:** Screen the data for anomalous values **Step 3:** Impute missing and anomalous values with the Multiple Imputation by Chained Equations (MICE) procedure **Step 4:** Distribute the imputation results to publication-ready files **Step 1**, **Step 2**, and **Step 4** are based on python code, were written in python^13^, and use the *pandas*^14^ and *numpy*^15,16^ packages. **Step 3** is written in the R programming language^11^ and relies on the *mice* package^10^. The versions of python, high-level python packages, R, and high-level R packages used in the analysis are: • python = 3.7.3 numpy = 1.16.2 pandas = 0.24.2 urllib3 = 1.24.1 • r-base = 3.5.1 r-mice = 3.6.0 r-dplyr = 0.7.6 r-data.table = 1.11.4 r-zoo = 1.8_3 parallel = 20200122 r-reshape2 = 1.4.3 r-markdown = 0.8 r-rmarkdown = 1.10 r-lubridate = 1.7.4 multiprocess = 0.70.9 The archived code used to clean and analyze the data is supplemented by an archived version of the Conda computing environment used for the analysis (ref. ^17^) with the corresponding DOIs: • v1.0 (specific version): 10.5281/zenodo.3736784 • Concept (all versions): 10.5281/zenodo.3736783 The data cleaning was run on Mac OSX 10.14.6. A complete list of every package present in the Conda computing environment can be found in the **package-list.txt** file in the code archive. The archived Conda environment can be used on Mac OSX systems circa 2020. Instructions for setting up the environment are included in both the code and computing environment archives. For other operating systems, we include an **environment.yml** file containing the high-level packages mentioned above. Instructions for setting up a Conda computing environment using the **environment.yml** file are also included in the code archive.
