# Supplementary material for: Developing reliable hourly electricity demand data through screening and imputation
Source: Sci Data. 2020 May 26;7:155. doi: 10.1038/s41597-020-0483-x (PMC7250876; doi:10.1038/s41597-020-0483-x)
Supplement: Supplementary file 1 — Supplementary Information [file 41597_2020_483_MOESM1_ESM.pdf]

Developing reliable hourly electricity demand data through screening and imputation: supplementary material

Contents

Data acquisition details . . . S.1
Anomalous value screening details . . . S.1
Second anomaly screening details . . . S.7
Energy system modeling details . . . S.9

Data acquisition details

The full BA name associated with each EIA code is listed in Supplementary Table S.4 along with their associated geographic region used for the regional aggregates.

Anomalous value screening details

This section contains additional details of the anomalous value screening process. It provides motivation for the screening algorithms and describes exactly how each is implemented.

Step 1

Negative or zero filter: The 'negative or zero' filter enforces $d_t > 0\,\mathrm{MW}$ for all hours. This filter screens the non-physically plausible negative values as well as the unlikely zero values. Nearly all of the zero values returned from the EIA database are intermittently inserted within 'normal' data. Zero appears to be a common choice of default value for some BAs when their standard reporting procedures lapse. It is technically possible for the electricity demand of an entire BA to reach zero; however, it is very unlikely. In cases of extreme weather induced power outages, we expect demand to trend downwards and then slowly recover rather than plunging to zero.

In the four years of analyzed data, we observe four cases with demand trending down to zero and then recovering. The two confirmed full-service-area outages are both from the NSB BA, a municipal electric utility serving approximately 26,000 customers in Florida (ref. [S.22]). These cases are caused by Hurricane Matthew in October 2016 and Hurrican Irma in September 2017 (Supplementary Figure S.7) (refs. [S.23, S.24]). No special treatment is given to these regions during screening or imputation; the imputed results are included in the cleaned data product like every other anomalous instance.

News posts from the SCL BA indicate their zero demand event was a false alarm during system reliability tests:

- 2:18 PM, 15 December 2016: "Power outage Fremont, Wallingford, U District, parts of Queen Anne; 20,128 customers out; cause unknown" (`https://twitter.com/seacitylight/status/809523148219645952`).
- 2:32 PM, 15 December 2016: "Outage was false alarm. No power lost" (`https://twitter.com/SEACityLight/status/809526584675811328`).

EIA Code	Balancing Authority Name	Region
AEC	PowerSouth Energy Cooperative	Southeast
AECI	Associated Electric Cooperative, Inc.	Midwest
AVA	Avista Corporation	Northwest
AZPS	Arizona Public Service Company	Southwest
BANC	Balancing Authority of Northern California	California
BPAT	Bonneville Power Administration	Northwest
CHPD	Public Utility District No. 1 of Chelan County	Northwest
CISO	California Independent System Operator	California
CPLE	Duke Energy Progress East	Carolinas
CPLW	Duke Energy Progress West	Carolinas
DOPD	PUD No. 1 of Douglas County	Northwest
DUK	Duke Energy Carolinas	Carolinas
EPE	El Paso Electric Company	Southwest
ERCO	Electric Reliability Council of Texas, Inc.	Texas
FMPP	Florida Municipal Power Pool	Florida
FPC	Duke Energy Florida, Inc.	Florida
FPL	Florida Power & Light Co.	Florida
GCPD	Public Utility District No. 2 of Grant County, Washington	Northwest
GVL	Gainesville Regional Utilities	Florida
HST	City of Homestead	Florida
IID	Imperial Irrigation District	California
IPCO	Idaho Power Company	Northwest
ISNE	ISO New England	New England
JEA	JEA	Florida
LDWP	Los Angeles Department of Water and Power	California
LGEE	Louisville Gas and Electric Company and Kentucky Utilities Company	Midwest
MISO	Midcontinent Independent System Operator, Inc.	Midwest
NEVP	Nevada Power Company	Northwest
NSB	Utilities Commission of New Smyrna Beach	Florida
NWMT	NorthWestern Corporation	Northwest
NYIS	New York Independent System Operator	New York
OVEC	Ohio Valley Electric Corporation	Mid-Atlantic
PACE	PacifiCorp East	Northwest
PACW	PacifiCorp West	Northwest
PGE	Portland General Electric Company	Northwest
PJM	PJM Interconnection, LLC	Mid-Atlantic
PNM	Public Service Company of New Mexico	Southwest
PSCO	Public Service Company of Colorado	Northwest
PSEI	Puget Sound Energy, Inc.	Northwest
SC	South Carolina Public Service Authority	Carolinas
SCEG	South Carolina Electric & Gas Company	Carolinas
SCL	Seattle City Light	Northwest
SEC	Seminole Electric Cooperative	Florida
SOCO	Southern Company Services, Inc. - Trans	Southeast
SPA	Southwestern Power Administration	Central
SRP	Salt River Project Agricultural Improvement and Power District	Southwest
SWPP	Southwest Power Pool	Central
TAL	City of Tallahassee	Florida
TEC	Tampa Electric Company	Florida
TEPC	Tucson Electric Power	Southwest
TIDC	Turlock Irrigation District	California
TPWR	City of Tacoma, Department of Public Utilities, Light Division	Northwest
TVA	Tennessee Valley Authority	Tennessee
WACM	Western Area Power Administration - Rocky Mountain Region	Northwest
WALC	Western Area Power Administration - Desert Southwest Region	Southwest
WAUW	Western Area Power Administration - Upper Great Plains West	Northwest

Table S.4: The full balancing authority name and the code used by the EIA for identification in their database are shown. The mapping of each balancing authority to the 13 EIA regions is also listed.

- 2:56 PM, 15 December 2016: “We test outage system for reliability. This was a rare false alarm during testing. Sorry for confusion.” (https://twitter.com/SEACityLight/status/809532553141088256).

Lastly, there is no confirmation of the observed full-service-area outage for the IID BA. Other outages are reported within a few days of the instance leading us to believe that the data are erroneous:

- 4:08 PM, 10 June 2018: “Power Outage- Salton City. Estimated restoration time is unknown; troubleshooter en route, updates to follow” (https://twitter.com/IIDatWork/status/1005949938268639232).
- 9:53 PM, 17 June 2018: “Power Outage- City of Holtville. Estimated restoration time is unknown; Troubleshooter en route, updates to follow.” (https://twitter.com/IIDatWork/status/1008573478264606720).

A total of 863 hours, or 0.0455% of the total data, are screened by the ‘negative or zero’ filter.

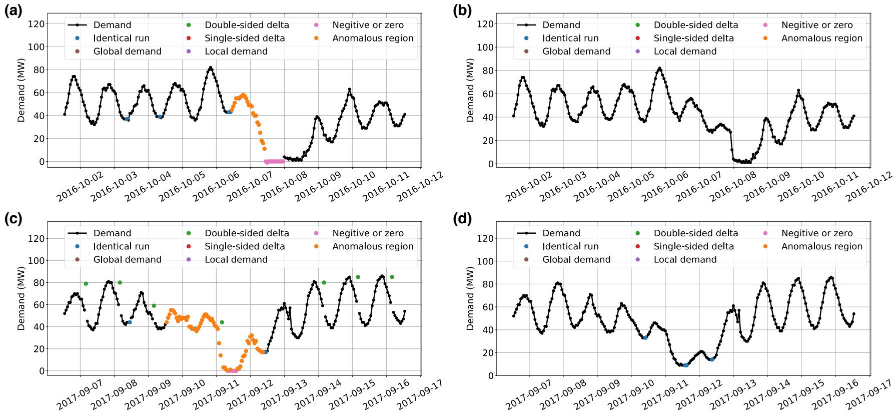

Figure S.7: The two confirmed full-service-area blackouts are shown. Both cases are for the NSB BA. The October 2016 example, (a) and (b), were during Hurricane Matthew while the September 2017 examples, (c) and (d), were during Hurricane Irma. Panels (a) and (c) show the original demand and value categorization, while (b) and (d) show the imputed results. No special treatment is given to these regions during imputation and the results can be seen in the right column.

Identical run filter: There are numerous instances where days or even months of reported demand values are identical. The ‘identical run’ filter screens out cases where the same value is reported for three or more consecutive hours. The filter marks the third hour onwards as anomalous; i.e. filter hour t if $d_{t-2} = d_{t-1} = d_t$. An example is seen in Figure 2 panel (a).

Because demand values are rounded to the nearest MW, it is possible for values to be identical hour-to-hour in accurately reported data. Repeated values are observed a non-negligible amount of time for the smaller BAs, particularly during the peaks and troughs of the daily demand cycle. The requirements of the ‘identical run’ filter strike a compromise between flagging false positives in the smaller BAs and removing every

single hour in long identical runs. The 'identical run' filter screens a total of 3,861 hours, or 0.204%, of the data.

Global demand filter: The 'global demand' filter screens demand values that are at least 10 times larger than the four year median demand value for each BA. The median demand value is calculated after applying the 'negative or zero' and 'identical run' filters. The threshold of ten times the median value was chosen to remove the most extreme upward outliers with the intention of the subsequent algorithms providing a more detailed removal of other upward outliers. The 'global demand' filter screens a total of 170 hours, or 0.00898% of the data.

Global demand ± 1 hour filter: Visual inspection of the data shows that the hours preceding and following hours screened by the 'global demand' filter are often upward outliers. If hour t is screened by the 'global demand' filter, hours $t-1$ and $t+1$ will be screened by the 'global demand ± 1 hour' filter. A total of 285 hours, or 0.0151% of the total data, are screened by this filter.

Step 2

Step 2 begins with a significantly cleaner data set because the four filters in **Step 1** have removed values that we are very confident are anomalous. At this point, we calculate variables for use in the subsequent screening algorithms.

Algorithm variables: Sub-weekly trends in the central tendency of the demand are represented using a 48 hour moving median variable (eq. S.8). Trends in $M_{t,48\mathrm{hr}}$ are likely related to the local weather.

$$M_{t,48\mathrm{hr}} = \mathrm{median}\left(d_{t-24}, d_{t-23}, \ldots\ d_{t+23}\right) \tag{S.8}$$

The $M_{t,48\mathrm{hr}}$ variable is resilient against sections of the data with several 'missing' and previously screened data values.

A longer 20 day version of the moving median value (eq. S.9) is used to track the seasonal demand cycle.

$$M_{t,480\mathrm{hr}} = \mathrm{median}\left(d_{t-240}, d_{t-239}, \ldots\ d_{t+239}\right) \tag{S.9}$$

The $M_{t,480\mathrm{hr}}$ is not intended to capture synoptic-scale weather changes.

The difference between d_t and $M_{t,48\mathrm{hr}}$ is denoted as $\Delta\left(d_t, M_{t,48\mathrm{hr}}\right) = d_t - M_{t,48\mathrm{hr}}$ and is used to understand the structure of the daily cycle. The typical spread in demand values about $M_{t,48\mathrm{hr}}$ is expressed as the IQR of $\Delta\left(d_t, M_{t,48\mathrm{hr}}\right)$ within a centered 10-day window (eq. S.10).

$$\begin{aligned} IQR_{dem,t} = \mathrm{IQR}\Big(&\Delta\left(d_{t-120}, M_{t-120,48\mathrm{hr}}\right), \\ &\Delta\left(d_{t-119}, M_{t-119,48\mathrm{hr}}\right), \ldots\ \Delta\left(d_{t+119}, M_{t+119,48\mathrm{hr}}\right)\Big) \end{aligned} \tag{S.10}$$

The $IQR_{dem,t}$ changes from season to season largely based on varying heating and cooling needs. The 10-day window provides enough data to estimate the IQR while remaining short enough to describe real synoptic-scale changes.

First-order differences between hours, $\delta\left(d_{t-1}, d_t\right) = d_{t-1} - d_t$, are used to identify outliers. The IQR of $\delta\left(d_{t-1}, d_t\right)$ quantifies the spread in values and is used to

set thresholds for screening demand values. We calculate the IQR of the first-order differences using a 10-day window (eq. S.11).

$$IQR_{\delta,t} = \mathrm{IQR}\Big(\delta\left(d_{t-120}, d_{t-119}\right), \delta\left(d_{t-119}, d_{t-118}\right), \ldots\ \delta\left(d_{t+118}, d_{t+119}\right)\Big) \tag{S.11}$$

We create a normalized local demand profile for the 24 hours in the daily cycle. For each hour of the day, we take the median $\Delta\left(d_t, M_{t,48\mathrm{hr}}\right)$ value over a centered 21-day window and normalize the result by the long-term median demand (eq. S.12).

$$h_{t,\mathrm{daily}} = \frac{1}{M_{t,480\mathrm{hr}}} \times median\Big(\Delta\left(d_{t-10\,days}, M_{t-10\,days,48\mathrm{hr}}\right), \Delta\left(d_{t-9\,days}, M_{t-9\,days,48\mathrm{hr}}\right), \ldots\ \Delta\left(d_{t+10\,days}, M_{t+10\,days,48\mathrm{hr}}\right)\Big) \tag{S.12}$$

$h_{t,\mathrm{daily}}$ is calculated over the large 21-day interval because it incorporates only a single value each day for each hour. When multiplied by $M_{t,48\mathrm{hr}}$, $(1 + h_{t,\mathrm{daily}})$ results in an estimate of the demand for any given hour based on local information. Demand values are compared against these estimates using eq. S.13.

$$r_t = \frac{d_t}{(1 + h_{t,\mathrm{daily}}) \times M_{t,48\mathrm{hr}}} \tag{S.13}$$

First-order differences between r_t values are denoted as $\delta\left(r_{t-1}, r_t\right)$ where $\delta\left(r_{t-1}, r_t\right) = r_{t-1} - r_t$. r_t is calculated as a ratio of the expected value making it and the derived quantity $\delta\left(r_{t-1}, r_t\right)$ relatively consistent throughout the year. Therefore, we calculate the IQR of $\delta\left(r_{t-1}, r_t\right)$ across the full four years of data (eq. S.14).

$$IQR_r = \mathrm{IQR\ for\ all\ } t \mathrm{\ in\ 4\ years} \left(\delta\left(r_{t-1}, r_t\right)\right) \tag{S.14}$$

Step 2 of the anomaly screening process consists of the following four screening algorithms that use the variables defined above.

Local demand filter: The 'local demand' filter screens values that deviate significantly from their expected values. The expected value is calculated as: $M_{t,48\mathrm{hr}} \times (1 + h_{t,\mathrm{daily}})$. Thresholds for deviations from the estimate are set in both the upwards and downwards directions. The thresholds are scaled by a determination of the typical spread in the daily cycle, $IQR_{dem,t}$. We screen demand values if either eq. S.15 or S.16 are true.

$$d_t > M_{t,48\mathrm{hr}} \times (1 + h_{t,\mathrm{daily}}) + IQR_{dem,t} \times 3.5 \tag{S.15}$$

$$d_t < M_{t,48\mathrm{hr}} \times (1 + h_{t,\mathrm{daily}}) - IQR_{dem,t} \times 2.5 \tag{S.16}$$

The 'local demand' filter screens a total of 671 hours, or 0.0354% of the data.

Double-sided delta filter: The 'double-sided delta' filter screens values that strongly violate the continuity of the demand data. The data typically exhibit relatively smooth transitions from one hour to the next. The filter targets single-hour spike-like features. We screen demand values if either equation S.17 or S.18 are true.

$$\delta\left(d_{t-1}, d_t\right) > IQR_{\delta,t} \times 2\ \&\ \delta\left(d_t, d_{t+1}\right) < IQR_{\delta,t} \times (-2) \tag{S.17}$$

$$\delta\left(d_{t-1}, d_t\right) < IQR_{\delta,t} \times (-2)\ \&\ \delta\left(d_t, d_{t+1}\right) > IQR_{\delta,t} \times 2 \tag{S.18}$$

There are many instances in the data where $\delta\left(d_{t-1}, d_t\right) > IQR_{\delta,t} \times 2$ or $\delta\left(d_{t-1}, d_t\right) < IQR_{\delta,t} \times (-2)$, such as the steeply rising shoulders of the daily cycle. These instances are not flagged by these criteria. The 'double-sided delta' filter screens a total of 1,212 hours, or 0.0640% of the data.

Single-sided delta filter: The 'single-sided delta' filter is designed to screen anomalous sequences of hours associated with large discontinuities, which collectively deviate significantly from their surrounding data. First-order differences in demand and r_t are used to identify discontinuities in the data. Deviations are defined based on the local values of $M_{t,48\text{hr}}$ and $M_{t,480\text{hr}}$. In general, $M_{t,48\text{hr}}$ represents the central tendency of the local data more accurately. However, when many anomalous values are present they can significantly alter $M_{t,48\text{hr}}$ and $M_{t,480\text{hr}}$ can provide a helpful benchmark.

A specific challenge for the 'single-sided delta' filter is that it must determine which demand values should be screened, the values preceding or following a large discontinuity. The 'single-sided delta' filter iterates over each hour twice, once in the forward direction, then again in the reverse direction. The algorithm specifically tracks the most recently iterated hour still labeled as 'okay', hour$_{okay}$, which is denoted with an *okay* subscript in the following description. Hour$_{okay}$ is initialized to the first 'okay' hour in the time series. For each hour, t, in each direction:

Step 1: Calculate the first-order difference in demand and r_t between hour$_{okay}$ and hour t: $\delta\left(d_{okay}, d_{\text{t}}\right)$ and $\delta\left(r_{okay}, r_{\text{t}}\right)$.

Step 2: Determine if the first-order differences are significant using IQR thresholds.

- If $\left|\delta\left(d_{okay}, d_{\text{t}}\right)\right| > IQR_{\delta,t} \times 5$ and $\left|\delta\left(r_{okay}, r_{\text{t}}\right)\right| > IQR_{r} \times 15$: continue to **Step 3**.
- Else: hour$_{okay}$ is incremented to hour t, proceed to **Step 1** for hour $t+1$.

Step 3: Determine which of the two hours deviate from their expected values the most using both 48 hour and 20 day moving medians. Screen the hour with the larger maximum deviation.

- If $\max\left(\left|1 - r_{okay}\right|, \left|1 - \frac{d_{okay}}{\left(1+h_{okay,\text{daily}}\right)\times M_{okay,480\text{hr}}}\right|\right) <$ $\max\left(\left|1 - r_{\text{t}}\right|, \left|1 - \frac{d_{\text{t}}}{\left(1+h_{\text{t},\text{daily}}\right)\times M_{\text{t},480\text{hr}}}\right|\right)$: screen hour t, hour$_{okay}$ remains unchanged, proceed to **Step 1** for hour $t+1$.
- Else: hour$_{okay}$ is incremented to hour t, proceed to **Step 1** for hour $t+1$.

The 'single-sided delta' filter screens a total of 181 hours, or 0.00960% of the data.

Anomalous region filter: There are multiple occurrences of especially chaotic segments of demand data. In some instances, the previous seven filters screen the entire chaotic segment. Other times, there are residual hours left unfiltered in the middle of multiple screened hours. We designed the 'anomalous region' filter with the intention of screening these remaining hours.

The algorithm focuses on the structure of 'okay' and 'missing' versus otherwise screened values. A few extra variables are calculated based on this structure. The percent of screened hours within ±24 hours of each hour t is calculated, $pct_{t,\text{screened}}$. If hour t is currently classified as 'okay', count the number of continuous 'okay' hours preceding and following giving the total length of 'okay' hours associated with hour t, $l_{t,okay}$.

For each hour, t:

Step 1: Determine if the number of screened values surrounding hour t is significant.

- If $pct_{t,\text{screened}} > 15\%$ (greater than 7 screened hours), proceed to **Step 2**.
- Else: proceed to **Step 1** for hour $t+1$.

Step 2: Iterate over the hours used to calculate $pct_{t,\text{screened}}$. For each hour t' in $(t-24, t-23, \dots\ t+24)$:

- If t' is not categorized as 'okay': continue to $t'+1$ in **Step 2** (t' is already screened or 'missing').
- If $l_{t',okay} > 24$ hours: continue to $t'+1$ in **Step 2** (t' is part of a substantial continuous section of 'okay' data).
- Else: screen t' and proceed to $t'+1$ in **Step 2**.

A prime example of the 'anomalous region' filter can be seen in panel (d) of Figure 2. The 'single-sided delta' and 'negative or zero' filters screen many hours in this example. But, there are some remaining anomalies undetected by other filters. The 'anomalous region' filter screens a total of 1,773 hours, or 0.0936% of the data.

Second anomaly screening details

Examples of the second two categories defined in the **Second anomaly screening** section are shown in Supplementary Figure S.8 for **Category 2** and Supplementary Figure S.9 for **Category 3**.

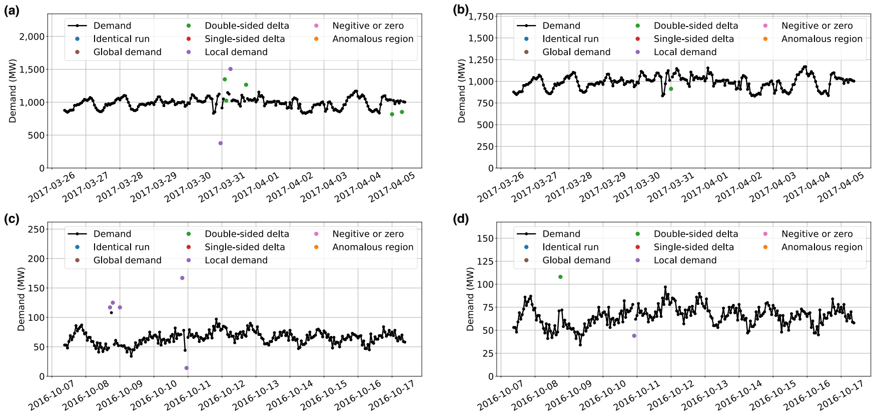

Figure S.8: These figures demonstrate two cases defined as **Category 2** where the original screening does not filter 100% of the anomalous values. Upon screening a second time, the remaining anomalous structures are identified and filtered. The two regions shown are the WALC BA, (a) and (b), and SPA BA (c) and (d). Panels (a) and (c) show the original demand and value categorization, while (b) and (d) show the results after the second screening.

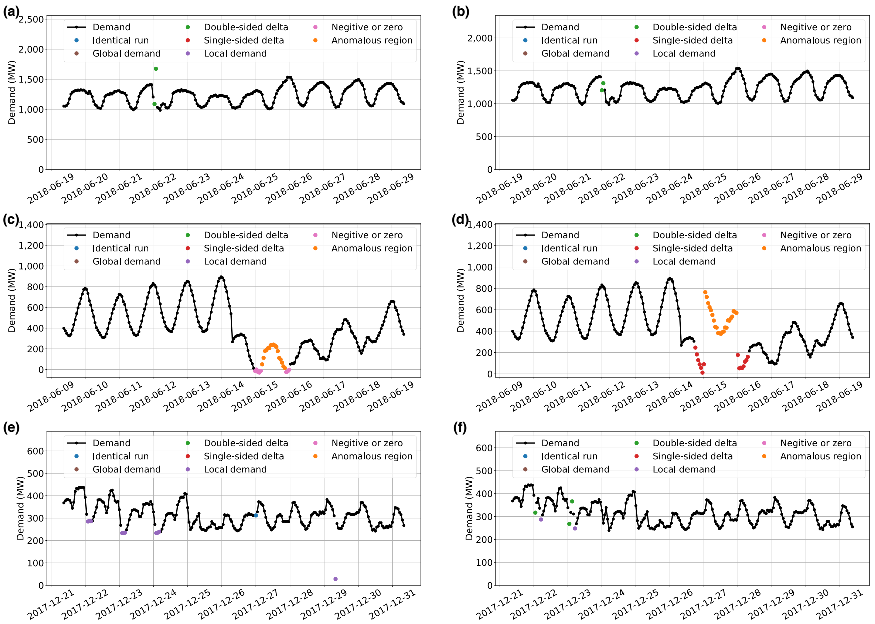

Figure S.9: These figures show the only three cases defined as **Category 3** where imputed values are flagged during the second screening. The examples shown are from the NWMT BA (a) and (b) and IID BA (c), (d), (e), and (f). In these examples, relatively stable daily cycles strongly influence the imputation process and pull the imputed values away from the non-flagged surrounding data leading to large discontinuities. Panels (a), (c), and (e) show the original demand and value categorization, while (b), (d), and (f) show the results after the second screening.

Energy system modeling details

The details of the **Energy system modeling** section are tabulated. Supplementary Table S.5 shows summary statistics including the mean, maximum, and minimum hourly demand for these three different example data cleaning methods. Supplementary Table S.6 shows the optmized system capacities for each region, for each technology case, and for each data cleaning method.

Region	Data Cleaning Mthd.	Mean (GW)	Max. (GW)	Max./Mean (%)	Min. (GW)	Min./Mean (%)
CONUS	Mthd. 1	455.3	3,768.8	828	218.0	48
	Mthd. 2	456.8	717.8	157	271.9	60
	Mthd. 3	458.5	717.5	156	309.0	67
Eastern	Mthd. 1	331.3	533.3	161	146.0	44
	Mthd. 2	332.5	533.7	161	169.1	51
	Mthd. 3	333.6	533.3	160	218.1	65
Western	Mthd. 1	83.1	3,419.6	4,116	37.0	45
	Mthd. 2	83.1	138.6	167	46.6	56
	Mthd. 3	83.6	139.4	167	59.3	71
Texas	Mthd. 1	40.9	73.3	179	0	0
	Mthd. 2	41.2	73.3	178	0	0
	Mthd. 3	41.3	73.6	178	25.1	61

Table S.5: Summary statistics including the mean, maximum, and minimum hourly demand for these different data cleaning techniques are shown for each geographic region for the modeled years, 2016 through 2018. A strong case for data cleaning is made when comparing the excessively large maximum demand values for **Method 1** in the Western and CONUS regions. Noticable differences are seen between **Method 2** and **Method 3** comparing the minimum demand values for Texas. The EIA imputation method (**Method 2**) lacks the ability to fill in long consecutive demand gaps.

Region	Included Tech-nologies	Data Cleaning Mthd.	Nat. Gas. (GW)	Capacities<br>Wind (GW)	Solar (GW)	Nuclear (GW)	Storage (GWh)
CONUS	all techs	Mthd. 1	3,769	0	0	0	0
		Mthd. 2	718	0	0	0	0
		Mthd. 3	717	0	0	0	0
CONUS	no NG	Mthd. 1	-	203	1,951	133	14,677
		Mthd. 2	-	480	282	420	574
		Mthd. 3	-	483	290	422	542
CONUS	no NG, no nuclear	Mthd. 1	-	604	2,030	-	14,638
		Mthd. 2	-	2,248	765	-	2,222
		Mthd. 3	-	2,269	757	-	2,233
Eastern	all techs	Mthd. 1	533	0	0	0	0
		Mthd. 2	534	0	0	0	0
		Mthd. 3	533	0	0	0	0
Eastern	no NG	Mthd. 1	-	76	113	399	461
		Mthd. 2	-	77	112	400	462
		Mthd. 3	-	77	106	403	445
Eastern	no NG, no nuclear	Mthd. 1	-	1,807	512	-	3,489
		Mthd. 2	-	1,766	514	-	3,755
		Mthd. 3	-	1,818	504	-	3,575
Western	all techs	Mthd. 1	3,420	0	0	0	0
		Mthd. 2	139	0	0	0	0
		Mthd. 3	139	0	0	0	0
Western	no NG	Mthd. 1	-	0	460	0	18,894
		Mthd. 2	-	42	66	84	190
		Mthd. 3	-	50	71	82	187
Western	no NG, no nuclear	Mthd. 1	-	0	460	-	18,894
		Mthd. 2	-	292	215	-	728
		Mthd. 3	-	290	216	-	733
Texas	all techs	Mthd. 1	73	0	0	0	0
		Mthd. 2	73	0	0	0	0
		Mthd. 3	74	0	0	0	0
Texas	no NG	Mthd. 1	-	0	12	54	97
		Mthd. 2	-	0	12	54	97
		Mthd. 3	-	0	12	54	97
Texas	no NG, no nuclear	Mthd. 1	-	101	275	-	754
		Mthd. 2	-	101	275	-	754
		Mthd. 3	-	101	275	-	754

Table S.6: Optimized system capacities are shown for each region, for the different technology cases, and for each data cleaning method. The capacities are for systems capable of meeting 100% of the electricity demand profile for each region. The "-" denote technologies that were excluded from the model configuration. Natural gas is abbreviated as NG.

References

[S.22] Fred Wilson & Associates, Inc. *Electric utility master plan 2016*. Utilities commission, City of New Smyrna Beach, Florida. https://www.ucnsb.org/sites/default/files/masterplan-v3-01302017.pdf, (2016).

[S.23] News 13 Florida. *Matthew: Central Florida power outages.* https://www.baynews9.com/fl/tampa/news/2016/10/6/central_florida_expe, (2016).

[S.24] Garland Power & Light. *GP&L work in Florida continues after hurricane Irma*. https://www.gpltexas.org/Home/Components/News/News/98/17?arch=1, (2017).
